# Increased care at discharge from COVID-19: The association between pre-admission frailty and increased care needs after hospital discharge; a multicentre European observational cohort study

**DOI:** 10.1186/s12916-020-01856-8

**Published:** 2020-12-18

**Authors:** A. Vilches-Moraga, A. Price, P. Braude, L. Pearce, R. Short, A. Verduri, M. Stechman, J. T. Collins, E. Mitchell, A. G. Einarsson, S. J. Moug, T. J. Quinn, B. Stubbs, K. McCarthy, P. K. Myint, J. Hewitt, B. Carter, on behalf of the COPE Study, Charlotte Davey, Sheila Jones, Kiah Lunstone, Alice Cavenagh, Louis Evans, Charlotte Silver, Thomas Telford, Rebecca Simmons, Tarik El Jichi Mutasem, Sandeep Singh, Dolcie Paxton, Will Harris, Norman Galbraith, Emma Bhatti, Jenny Edwards, Siobhan Duffy, Joanna Kelly, Caroline Murphy, Carly Bisset, Ross Alexander, Madeline Garcia, Shefali Sangani, Thomas Kneen, Thomas Lee, George Kyriakopoulos, Michael Thomas, Denise Tan, Enrico Clini, Eilidh Bruce, Frances Rickard, Fenella Balow-Pay, James Hesford, Mark Holloway

**Affiliations:** 1 Faculty of Medical and Human Services, University of Manchester, Manchester, England; 2 Salford Royal Hospital Foundation Trust, Salford, England; 3 North Bristol NHS Trust, Bristol, England; 4 Department of Biostatistics and Health Informatics, King’s College London, London, England; 5 University Hospital of Modena Policlinico, University of Modena and Reggio Emilia, Modena, Italy; 6 University Hospital of Wales, Cardiff University, Cardiff, Wales; 7 Ysbyty Ystrad Fawr, Aneurin Bevan University Health Board, Newport, Wales; 8 NHS Grampian, Aberdeen, UK; 9 Royal Alexandra Hospital, Paisley, Scotland; 10 Glasgow Royal Infirmary, Glasgow, Scotland; 11 Physiotherapy Department, South London and Maudsley NHS Foundation Trust, Denmark Hill, London, UK; 12 University of Aberdeen, Aberdeen, Scotland; 13 Aneurin Bevan Health Board, Cardiff University, Cardiff, Wales

**Keywords:** COVID-19, Clinical frailty scale, Care need, Discharge destination, Frailty, Older people, Increased care need

## Abstract

**Background:**

The COVID-19 pandemic has placed significant pressure on health and social care. Survivors of COVID-19 may be left with substantial functional deficits requiring ongoing care. We aimed to determine whether pre-admission frailty was associated with increased care needs at discharge for patients admitted to hospital with COVID-19.

**Methods:**

Patients were included if aged over 18 years old and admitted to hospital with COVID-19 between 27 February and 10 June 2020. The Clinical Frailty Scale (CFS) was used to assess pre-admission frailty status. Admission and discharge care levels were recorded. Data were analysed using a mixed-effects logistic regression adjusted for age, sex, smoking status, comorbidities, and admission CRP as a marker of severity of disease.

**Results:**

Thirteen hospitals included patients: 1671 patients were screened, and 840 were excluded including, 521 patients who died before discharge (31.1%). Of the 831 patients who were discharged, the median age was 71 years (IQR, 58–81 years) and 369 (44.4%) were women. The median length of hospital stay was 12 days (IQR 6–24). Using the CFS, 438 (47.0%) were living with frailty (≥ CFS 5), and 193 (23.2%) required an increase in the level of care provided. Multivariable analysis showed that frailty was associated with an increase in care needs compared to patients without frailty (CFS 1–3). The adjusted odds ratios (aOR) were as follows: CFS 4, 1.99 (0.97–4.11); CFS 5, 3.77 (1.94–7.32); CFS 6, 4.04 (2.09–7.82); CFS 7, 2.16 (1.12–4.20); and CFS 8, 3.19 (1.06–9.56).

**Conclusions:**

Around a quarter of patients admitted with COVID-19 had increased care needs at discharge. Pre-admission frailty was strongly associated with the need for an increased level of care at discharge. Our results have implications for service planning and public health policy as well as a person's functional outcome, suggesting that frailty screening should be utilised for predictive modelling and early individualised discharge planning.

## Background

COVID-19 has resulted in large numbers of people being admitted to hospital worldwide [1–3]. The UK government guidance published on 25 August 2020 estimates that 95% of patients admitted with COVID-19 may be discharged home, but 50% would require voluntary and community support and 45% health and social care services, and 4% would be discharged to rehabilitation and 1% into long-term care facilities [4]. However, these figures do not take into account those that already have these services in place pre-admission. Patients may experience functional deterioration requiring temporary or permanent support from community social care and rehabilitation services on discharge from hospital [5–7]. A proportion of this group may require a change in living situation, for example, a long-term care facility such as residential or nursing home.

People at higher risk of requiring hospital admission due to COVID-19 are older with greater levels of multimorbidity and frailty [8, 9]. Frailty represents increased vulnerability to stressors due to declined physiological systems and loss of homeostasis [10, 11]. In a wide range of conditions, even after adjusting for age and comorbidity, frailty has been reported as an independent predictor of mortality, prolonged hospital stay, and increased care needs following hospital discharge [12, 13]. Similar effects due to COVID-19 have been reported including mortality and increased length of hospital stay [9].

To date, most studies in older people with COVID-19 have focussed on mortality with little attention to functional outcomes. However, in older adults, mortality is not always the most important outcome. Previous studies assessing the impact of frailty on quality of life in other conditions have demonstrated that for older adults, independent living is a more important outcome than death. A higher value is placed on continuing day-to-day societal roles, reducing the risk of isolation and loneliness and avoiding poor future health outcomes [14–16]. Currently, there is no literature describing the factors associated with a loss of independence, or associated increase in care needs, after hospital admission post-acute COVID-19. The aim of this study was to investigate the association between pre-admission frailty and change in the level of care needs on discharge from hospital in patients admitted with COVID-19.

## Methods

### Study design

Data were obtained as part of a multicentre observational study: COPE (COVID-19 in Older People study). The study was authorised by the Health Research Authority (20/HRA/1898) in the UK and the Ethics Committee of Policlinico Hospital Modena (Reference 369/2020/OSS/AOUMO) in Italy. The full study details can be found within the COPE protocol [17], and the main study findings are reported elsewhere [9]. This manuscript follows the STROBE statement for reporting of cohort studies. Investigators carried out standardisation training in both data collection and CFS assessment. A central MACRO database, hosted by King’s Clinical Trials Unit (KCTU), was used to enter data centrally.

### Setting

The COPE-Discharge study used an established network of twelve UK sites and one Italian site. The UK centres included Ysbyty Ystrad Fawr in Caerphilly, Royal Gwent Hospital in Newport, Nevill Hall Hospital in Abergavenny, University Hospital of Wales in Cardiff, Southmead Hospital in Bristol, Aberdeen Royal Infirmary, Royal Alexandra Hospital in Paisley, Inverclyde Royal Hospital, Salford Royal Hospital, Glasgow Royal Infirmary, Maidstone Hospital, and Ysbyty Gwynedd in Bangor. The Italian centre was the University Hospital of Modena Policlinico.

### Participants

Each site research team screened hospital admission lists daily. The ethical approval was such that formal written consent from participants was deemed as not being required as all data were routinely collected in hospital records.

#### Inclusion/exclusion criteria

The study included consecutive hospitalised patients aged 18 years or older with a confirmed diagnosis of COVID-19 admitted between 27 February and 10 June 2020; diagnostic criteria included laboratory-confirmed SARS-CoV-2-positive swab or a clinical diagnosis of COVID-19 based on signs, symptoms, and supporting radiology. Patients were excluded due to missing care level at admission and discharge, were not discharged from hospital, and that died prior to discharge*.*

### Outcome

The primary outcome was increased care needs at discharge. Care was recorded as an ordinal variable, with seven levels of increasing dependence care needs: at home without formal carers, own home with carers (formal or informal), intermediate care, increased number of daily carer visits, sheltered care, residential home, and nursing home.

The number of daily carer visits required by patients was measured at admission and discharge*.* Sheltered care was the accommodation of private independent units with shared facilities such as gardens and lounges and a warden on site. Residential care was defined as 24-h supported care managed by non-nursing trained care staff. Nursing care comprised service users receiving 24-h support from nursing staff, requiring assistance with most personal daily activities, or support with complex physical and/or psychological needs. Intermediate care varied depending on local provision and was defined as short-term care (either in an institution or individual’s home) designed to facilitate the transition from hospital to home [18, 19]. Intermediate care services develop person-centred goals aimed at optimising independence and well-being of individuals through collaborative multidisciplinary holistic assessment and interventions.

### Covariates

Demographic and clinical characteristics recorded at admission were age, sex, smoking status (never, previous, or current), C-reactive protein (CRP) as a marker of disease severity, estimated glomerular filtration rate (eGFR), previous history of coronary artery disease (CAD), diabetes mellitus, chronic obstructive pulmonary disease (COPD), and hypertension (no, yes not on treatment, and yes on treatment).

Frailty was scored based on a functional status history from 2 weeks prior to admission and was measured using the Clinical Frailty Scale (CFS). The CFS is a 9-point score, from 1 being very fit, 2 well, 3 managing well, 4 living with very mild frailty, 5 living with mild frailty, 6 living with moderate frailty, 7 living with severe frailty, 8 living with very severe frailty, and 9 terminally ill but otherwise living with severe frailty [13]. For the purpose of the analyses, CFS categories 1–3 were grouped and used as a reference group. In each site, the assessment of CFS in patients was undertaken by clinical teams comprising a combination of consultant geriatricians, emergency physicians, and intensive care consultants. For all COVID-19 patients admitted to hospital, the CFS was documented in a dedicated section on the admission notes. To safeguard data quality, each principal investigator ensured adequate knowledge within the data collection team of frailty scoring.

### Statistical analysis

We analysed the change in care level using a mixed-effects logistic regression, fitted with a random effect model to account for variation occurring at each hospital site. Care level was associated with baseline frailty and adjusted for patient age group (< 65, 65–79, ≥ 80 years old), sex, smoking status (never smoker, ex-smoker, current smoker), CRP (≥ 40 mg/L taken as abnormal), diabetes (no/yes), hypertension (no/yes/yes and on treatment), coronary artery disease (no/yes), and reduced renal function (eGFR < 60, ≥ 60 mL/min/1.73 m^2^). Both crude odds ratios (OR) and adjusted odds ratios (aOR) were calculated with 95% confidence intervals (95% CI). The analysis was carried out using Stata version 15.

We carried out a sensitivity analysis to assess longer-term increased dependence by excluding patients that were discharged with intermediate care.

## Results

**Fig. 1:**
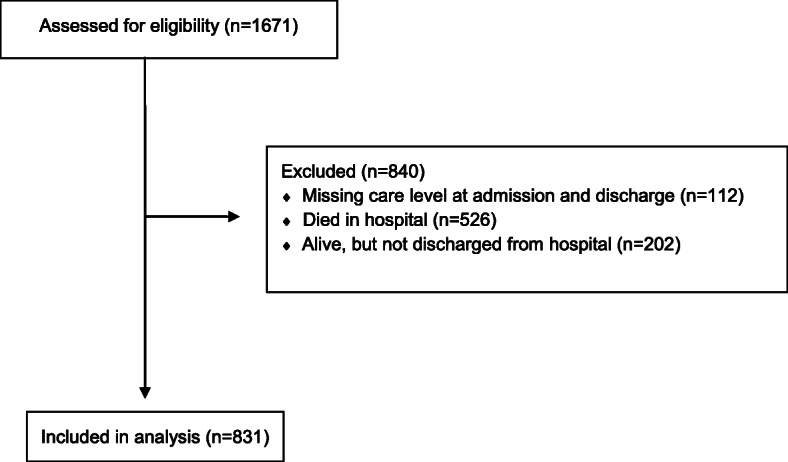
CONSORT flow diagram

As per the CONSORT flow diagram (Fig. 1), a total of 1671 patient records were entered into the COPE-Discharge database from 13 sites. Eight hundred and forty patients (840) were excluded: 112 patients due to missing care level at admission and discharge, 202 patients due to being alive still in hospital, and 526 had died in hospital.

Of the 831 patients meeting the inclusion criteria (Table 1), the median patient age was 71 years (58–81 [IQR], range 19 to 100) and 369 (44.4%) were women. The median length of hospital stay was 12 days (6–24 [IQR], 0–145 [min–max]), and 388 (46.7%) patients were considered to be living with frailty (CFS 5 to 9). Of the included patients, 30 (3.6%) were under 65 years old and had an increased level of care. Of the 30 patients, 9 went from living independently at admission to requiring a carer, and 13 required intermediate care. There were 36 patients with a missing care level at discharge imputed as not experiencing an increased care.

**Table 1:** Patient characteristics, by increased level of care at discharge following hospital admission of adult patients hospitalised with COVID-19

	Care level at discharge (compared to admission)		Total
	The same level of care at discharge	An increased level of care at discharge	
**Sites**	**(*n*** **= 638)**	**(*n*** **= 193)**	**(*n*** **= 831)**
Hospital A	58 (56.3)	45 (43.7)	103 (12.4)
Hospital B	73 (77.7)	21 (22.3)	94 (11.3)
Hospital C	32 (100)	0 (0)	32 (3.9)
Hospital D	47 (61.8)	29 (38.2)	76 (9.1)
Hospital E	14 (28.6)	35 (71.4)	49 (5.9)
Hospital F	183 (86.7)	28 (13.3)	211 (25.4)
Hospital G	17 (94.4)	1 (5.6)	18 (2.2)
Hospital H	85 (88.5)	11 (11.5)	96 (11.6)
Hospital I	79 (87.8)	11 (12.2)	90 (10.8)
Hospital J	17 (68.0)	8 (32.0)	25 (3.0)
Hospital K	6 (75.0)	2 (25.0)	8 (1.0)
Hospital L	14 (100)	0 (0)	14 (1.7)
Hospital M	13 (86.7)	2 (13.3)	15 (1.8)
**Age**			
Under 65 years	285 (90.5)	30 (9.5)	315 (37.9)
65 to 79 years	198 (72.3)	76 (27.7)	274 (33.0)
80 years or older	155 (64.0)	87 (36.0)	242 (29.1)
**Sex**			
Female	280 (75.9)	89 (24.1)	369 (44.4)
Male	357 (77.4)	104 (22.6)	461 (55.5)
Missing	1	0	1
**Smoking status**			
Never smokers	346 (76.5)	106 (23.4)	452 (54.4)
Ex-smokers	226 (75.3)	74 (24.7)	300 (36.1)
Current smokers	49 (80.3)	12 (19.7)	61 (7.3)
Missing	17	1	18
**Diabetes**			
No	481 (77.0)	144 (23.0)	625 (75.2)
**Yes**	156 (76.1)	49 (23.9)	205 (24.7)
Missing	1	0	1
**Hypertension**			
No	341 (78.8)	92 (21.2)	433 (52.1)
**Yes (not on treatment)**	70 (82.4)	15 (17.6)	164 (10.2)
**Yes (on treatment)**	227 (72.5)	86 (27.5)	313 (37.7)
**Coronary artery disease**			
No	521 (76.3)	162 (23.7)	683 (82.2)
Yes	117 (79.1)	31 (20.9)	148 (17.8)
**Elevated CRP (≥ 40)**			
No	205 (71.2)	83 (28.8)	288 (34.7)
Yes	433 (79.7)	110 (20.3)	543 (65.3)
**Renal function (eGFR < 60)**			
No	440 (77.5)	128 (22.5)	568 (68.4)
Yes	180 (73.5)	65 (26.5)	245 (29.5)
Missing	18	0	18
**Care level at admission**			
House/flat (no carer)	452 (75.8)	144 (24.2)	596 (71.7)
House/flat (with carer)	58 (78.4)	16 (21.6)	74 (8.9)
Sheltered care	27 (73.0)	10 (27.0)	37 (4.5)
Residential care	48 (85.7)	8 (14.3)	56 (6.7)
Nursing care	53 (81.5)	12 (18.5)	65 (7.8)
Missing	0	3	3
**Clinical Frailty Scale (CFS)**			
1, Very fit	53 (89.8)	6 (10.2)	59 (7.1)
2, Fit	104 (87.4)	15 (12.6)	119 (14.3)
3, Managing well	139 (86.9)	21 (13.1)	160 (19.3)
4, Vulnerable	80 (80.0)	20 (20.0)	100 (12.0)
5, Mildly frail	70 (63.1)	41 (36.9)	111 (13.4)
6, Moderately frail	72 (61.5)	45 (38.5)	117 (14.1)
7, Severely frail	99 (73.9)	35 (26.1)	134 (16.1)
8, Very severely frail	14 (63.6)	8 (36.4)	22 (2.6)
9, Terminally ill	3 (75.0)	1 (25.0)	4 (0.5)
Missing	4	1	5

Before hospital admission, 596 patients (71.7%) lived independently at home without a carer and a further 74 (8.9%) lived at home with carer support. The remaining 19.1% were living in either a sheltered, residential, or nursing care setting (Table 2). Across the 13 sites, 193 (23.2%) patients were discharged with an increase in care level compared with that documented on admission. Fewer patients under 65 years old (30 out of 315, 9.5%) required an increase in care level compared with those of aged 80 years or older (87/242, 35.9%).

**Table 2:** Care level at admission (rows) versus discharge care status (column) of adult patients hospitalised with COVID-19

	Care level at discharge								
	Missing	Own home	Own home (with carer visits)	Sheltered care	Residential care	Nursing care	Increased number of daily carer visits	Intermediate care	Total
**Care level on admission**									
Missing								3*	3
Own home	31	422	39*	1*	20*	19*	12*	52*	596
Own home (with carer visits)		9	48				11*	6*	74
Sheltered care	1	2		24			3*	7*	37
Residential care	2		1		45	6*		2*	56
Nursing care	2			1	1	49		12*	65
Total	36	433	88	26	66	74	26	82	831

*Data in bold indicate patients that were recorded as having increased care at discharge

As shown in Table 2, of the 596 patients who were living at home without formal care before admission, 143 (24.0%) required an increased level of care at discharge. Of the 74 patients who were living at home with carers before admission, 17 (23.0%) required an increased level of care. Of the 158 patients who came from a sheltered accommodation, residential, or nursing home, 30 (19.0%) required an increased level of care. An increased care level occurred in 10.2%, 12.6%, and 13.1% for the least frail categories of CFS 1, 2, and 3, respectively (Table 1). This compared to the following: CFS 4, 20.0%; CFS 5, 36.9%; CFS 6, 38.5%; CFS 7, 26.1%; and CFS 8, 36.4%.

In the crude logistic regression analysis, CFS was associated with an increase in care at discharge (Table 3). Compared to CFS 1–3, CFS 4 OR = 2.71 (95% CI 1.38–5.34, *p* = 0.004), CFS 5 OR = 5.63 (95% CI 3.08–10.27, *p* < 0.0001), CFS 6 OR = 5.90 (95% CI 3.24–10.75, *p* < 0.0001), CFS 7 OR = 3.50 (95% CI 1.90–6.47, *p* < 0.0001), and CFS 8 OR = 4.30 (95%CI 1.53–12.09, *p* = 0.006). The covariates associated with an increased level of care at discharge were age (compared to < 65 years: 65–79 OR = 3.71 (95% CI 2.18–6.30, *p* < 0.0001); ≥ 80 OR = 5.79 (95% CI 3.35–9.98, *p* < 0.0001)) and elevated CRP (≥ 40 mg/L) OR = 0.65 (95% CI 0.45–0.94, *p* = 0.022).

**Table 3:** Increased care at discharge of patients hospitalised with COVID-19

	Crude odds ratio (OR)		Adjusted OR (aOR)^**&**^ (***n*** = 810)^**&&**^	
	OR (95%CI)	*p* value	aOR (95%CI)	*p* value
**Age**				
Under 65	Reference Category		Reference Category	
65 to 79	3.71 (2.18–6.30)	< 0.0001	2.82 (1.57–5.06)	0.0005
≥ 80	5.79 (3.35–9.98)	< 0.0001	3.87 (2.07–7.26)	< 0.0001
**Sex** (female)	Reference Category		Reference Category	
Male	0.98 (0.68–1.40)	0.91	1.20 (0.81–1.79)	0.37
**Smoking status** (never)	Reference Category		Reference Category	
Ex-smokers	1.38 (0.95–2.02)	0.09	1.08 (0.71–1.63)	0.73
Current smokers	0.92 (0.45–1.90)	0.83	1.05 (0.47–2.33)	0.90
**Elevated CRP** (≥ 40)	0.65 (0.45–0.94)	0.022	0.73 (0.49–1.09)	0.12
**Patients with diabetes**	1.12 (0.75–1.69)	0.57	0.99 (0.63–1.55)	0.97
**Patients with CAD**	0.82 (0.51–1.31)	0.41	0.49 (0.29–0.82)	0.007
**Patients with hypertension**				
Yes (not on treatment)	0.82 (0.43–1.59)	0.56	0.65 (0.32–1.32)	0.23
Yes and on treatment	1.12 (0.77–1.63)	0.57	0.98 (0.64–1.50)	0.94
**Patients with reduced renal function (eGFR < 60)**	1.39 (0.95–2.03)	0.09	1.03 (0.67–1.58)	0.90
**Clinical Frailty Scale** (CFS 1 to 3)	Reference Category		Reference Category	
CFS 4	2.71 (1.38–5.34)	0.004	1.99 (0.97–4.11)	0.062
CFS 5	5.63 (3.08–10.27)	< 0.0001	3.77 (1.94–7.32)	0.0001
CFS 6	5.90 (3.24–10.75)	< 0.0001	4.04 (2.09–7.82)	< 0.0001
CFS 7	3.50 (1.90–6.47)	< 0.0001	2.16 (1.12–4.20)	0.022
CFS 8	4.30 (1.53–12.09)	0.006	3.19 (1.06–9.56)	0.039
CFS 9^&&&^				

^&^The multivariable mixed-effects logistic regression was adjusted for age group, sex, smoking, CRP, diabetes, CAD, hypertension, renal function, care level of admission, and the Clinical Frailty Scale

^&&^Twenty-one observations were excluded due to having missing covariate data

^&&&^The four patients that were terminally ill (CFS=9) were not included due to the low number of cases

Of the 831 included participants, 810 (97.5%) exhibited complete data and were included in the mixed-effects multivariable logistic regression analysis. After adjustment for the other comorbidities and CRP on admission, an increase in the level of care at discharge was associated with frailty. Compared to CFS 1–3, CFS 4 aOR = 1.99 (95% CI 0.97–4.11, *p* = 0.062), CFS 5 aOR = 3.77 (95% CI 1.94–7.32, *p* = 0.0001), CFS 6 aOR = 4.04 (95% CI 2.09–7.82, *p* < 0.0001), CFS 7 aOR = 2.16 (95% CI 1.12–4.20, *p* = 0.022), and CFS 8 aOR = 3.19 (95% CI 1.06–9.56, *p* = 0.039). Other covariates associated with an increased level of care were age (compared to < 65 years: 65–79 aOR = 2.82 (95% CI 1.57–5.06, *p* = 0.0005); > 80 aOR = 3.87 (95% CI 2.07–7.26, *p* < 0.0001)) and CAD (aOR = 0.49, 95% CI 0.29–0.82, *p* = 0.007).

A sensitivity analysis carried out that only included patients that received a longer-term increase in care (by removing intermediate care) at discharge found frailty was associated with an increased care needs, compared to CFS 1–3.

## Discussion

Our study has demonstrated that pre-admission frailty status is associated with a person’s level of independence after discharge from hospital following admission with COVID-19. Although many patients had no formal care prior to admission, almost a quarter acquired COVID-19-related functional dependence exhibited by additional care required on discharge. These findings are in keeping with other studies demonstrating worse functional outcomes associated with greater pre-hospital levels of frailty [20].

Although not synonymous with advancing age, frailty is more prevalent in the older population, and as a consequence, older people are more likely to require care. These data have shown that risk of increased care needs following hospitalisation with COVID-19 is also associated with increasing age, with those over 80 years or older significantly more likely to require increased care at discharge than younger people.

The association of frailty and age with increased functional dependence on discharge can be used to assist with early identification, at the point of hospital admission, of people at risk of needing more care. These data can aid clinicians in providing early tailored assessments and management plans to patients. One paradigm of this tailored assessment is a comprehensive geriatric assessment (CGA) effected through geriatric medicine clinicians in conjunction with the multidisciplinary team. CGA produces a personalised and integrated management plan using a multimodal, multimorbidity, and multidisciplinary approach over time. It has been shown to reduce post-acute long-term functional dependence and mortality [21–23]. Ensuring CGA-trained clinicians are embedded in acute hospital pathways will allow early screening of vulnerable patients and the potential chance to modify post-COVID outcomes.

These data may be unique to hospital admissions during the pandemic and not pertain to non-COVID times. Hospital-based teams are being faced with unique challenges: streamlined discharge processes aimed at maintaining capacity within the acute setting, reduced access to specialist inpatient therapy resources due to high service demand, sickness-related staff absences, and the breakdown of pre-pandemic informal care arrangements due to social distancing restrictions [4]. It is difficult to interpret if these data reflect the Department of Health’s estimate that 50% of patients would be able to be discharged home without care—50.7% in this cohort returned to their own home with no support, but this figure excludes the 19% in this study admitted from sheltered accommodation or a care home—and whether the Department of Health estimate includes these groups [4]. Furthermore, these findings have implications for both health and social care funding with a large economic package required to provide adequate support for a more dependent population post COVID-19. Local providers can use these data to anticipate short-term budgets based on the frailty statuses of the hospitalised population. Future research should focus on the trajectory of patients' COVID-19-acquired functional dependence, as well as examine population-based frailty scoring with the same associations of an increased level of care, in order to improve the regional and national social care predicted spend [4].

To minimise the personal and societal burden of COVID-19-related functional decline and increased care needs, urgent strategies are required: firstly, avoiding transmission of the virus through public health techniques such as contact tracing, infection control measures, and social distancing including shielding; secondly, modification of frailty pre-admission may ameliorate the risk of post-acute deterioration [24–28]; thirdly, post-acute rehabilitation through specialist multidisciplinary rehabilitation services needs to be targeted at those who have been affected to promote a return to pre-admission function [29–31].

### Strengths and limitations

This is a multicentre study with a large sample size receiving real-world care. Data were collected and curated by clinicians with an interest in older adults and processes aimed at maximising data completion and decreasing bias. Baseline patient characteristics and clinical outcomes demonstrated are in line with other COVID-19 publications suggesting external validity, for example, almost three quarters of patients in this cohort were 65 years of age or older consistent with published evidence [1–3].

This study had several limitations: data to be cautious include the inter-hospital consistency of the services delivered within the term intermediate care; the difference between crude and adjusted effect of CAD; and the use of the CSF in people of all ages as this tool has been primarily validated in populations over 65 years. This study describes an increased care level at the time of hospital discharge but not beyond that time.

## Conclusions

Frailty is associated with an increased level of care post hospital admission with COVID-19. In this study, 23.2% of discharged patients required an increased care level on discharge. This study suggests that future public health approaches must take into account a large number of patients with increased care needs and position adequate resources to ensure robust supported discharge schemes for those admitted to hospital. Frailty screening should become a standard practice at admission in order to identify patients early who are most likely to benefit from multidisciplinary health and social care attention.

## Data Availability

Data is available on request from the corresponding author on receipt of a statistical analysis plan addressing an important new scientific question, approved by the COPE Study Steering Committee.
